# Development of a measuring app for systemic sclerosis-related digital ulceration (SALVE: Scleroderma App for Lesion VErification)

**DOI:** 10.1093/rheumatology/keae371

**Published:** 2024-07-19

**Authors:** Adrian K Davison, Ashma Krishan, Robert P New, Andrea Murray, Graham Dinsdale, Joanne Manning, Frances Hall, John D Pauling, Andy Vail, Kathryn Kearney, Helen Patrick, Michael Hughes, William Dixon, Mark Dickinson, Chris Taylor, Ariane L Herrick

**Affiliations:** Centre for Musculoskeletal Research, The University of Manchester, Northern Care Alliance NHS Foundation Trust, Manchester Academic Health Science Centre, Manchester, UK; Department of Computing and Mathematics, Manchester Metropolitan University, Manchester, UK; Centre for Biostatistics, The University of Manchester, Northern Care Alliance NHS Foundation Trust, Manchester Academic Health Science Centre, Manchester, UK; Centre for Musculoskeletal Research, The University of Manchester, Northern Care Alliance NHS Foundation Trust, Manchester Academic Health Science Centre, Manchester, UK; Centre for Musculoskeletal Research, The University of Manchester, Northern Care Alliance NHS Foundation Trust, Manchester Academic Health Science Centre, Manchester, UK; Centre for Musculoskeletal Research, The University of Manchester, Northern Care Alliance NHS Foundation Trust, Manchester Academic Health Science Centre, Manchester, UK; Northern Care Alliance NHS Foundation Trust, Salford, UK; Centre for Musculoskeletal Research, The University of Manchester, Northern Care Alliance NHS Foundation Trust, Manchester Academic Health Science Centre, Manchester, UK; Northern Care Alliance NHS Foundation Trust, Salford, UK; Cambridge University Hospitals NHS Foundation Trust, Cambridge, UK; Department of Rheumatology, North Bristol NHS Trust, Bristol, UK; Musculoskeletal Research Unit, Translational Health Sciences, Bristol Medical School, University of Bristol, Bristol, UK; Centre for Biostatistics, The University of Manchester, Northern Care Alliance NHS Foundation Trust, Manchester Academic Health Science Centre, Manchester, UK; Centre for Musculoskeletal Research, The University of Manchester, Northern Care Alliance NHS Foundation Trust, Manchester Academic Health Science Centre, Manchester, UK; Centre for Musculoskeletal Research, The University of Manchester, Northern Care Alliance NHS Foundation Trust, Manchester Academic Health Science Centre, Manchester, UK; Centre for Musculoskeletal Research, The University of Manchester, Northern Care Alliance NHS Foundation Trust, Manchester Academic Health Science Centre, Manchester, UK; Northern Care Alliance NHS Foundation Trust, Salford, UK; Centre for Musculoskeletal Research, The University of Manchester, Northern Care Alliance NHS Foundation Trust, Manchester Academic Health Science Centre, Manchester, UK; Photon Science Institute, The University of Manchester, Manchester, UK; Centre for Imaging Sciences, Division of Informatics, Imaging & Data Sciences, The University of Manchester, Manchester, UK; Centre for Musculoskeletal Research, The University of Manchester, Northern Care Alliance NHS Foundation Trust, Manchester Academic Health Science Centre, Manchester, UK; Northern Care Alliance NHS Foundation Trust, Salford, UK

**Keywords:** digital ulcers, outcome measure, smartphone photography, smartphone app, systemic sclerosis

## Abstract

**Objectives:**

To test the hypothesis that photographs (in addition to self-reported data) can be collected daily by patients with SSc using a smartphone app designed specifically for digital lesions, and could provide an objective outcome measure for use in clinical trials.

**Methods:**

An app was developed to collect images and patient-reported outcome measures including Pain score and the Hand Disability in Systemic Sclerosis-Digital Ulcers (HDISS-DU) questionnaire. Participants photographed their lesion(s) each day for 30 days and uploaded images to a secure repository. Lesions were analysed both manually and automatically, using a machine learning approach.

**Results:**

Twenty-five patients with SSc-related digital lesions consented, of whom 19 completed the 30-day study, with evaluable data from 27 lesions. Mean (s.d.) baseline Pain score was 5.7 (2.4) and HDISS-DU 2.2 (0.9), indicating high lesion- and disease-related morbidity. A total of 506 images were used in the analysis [mean number of used images per lesion 18.7 (s.d. 8.3)]. Mean (s.d.) manual and automated lesion areas at day 1 were 11.6 (16.0) and 13.9 (16.7) mm^2^, respectively. Manual area decreased by 0.08 mm^2^ per day (2.4 mm^2^ over 30 days) and automated area by 0.1 mm^2^ (3.0 mm^2^ over 30 days). Average gradients of manual and automated measurements over 30 days correlated strongly (r = 0.81). Manual measurements were on average 40% lower than automated ones, with wide limits of agreement.

**Conclusion:**

Even patients with significant hand disability were able to use the app. Automated measurement of finger lesions could be valuable as an outcome measure in clinical trials.

## Graphical Abstract

**Figure:**
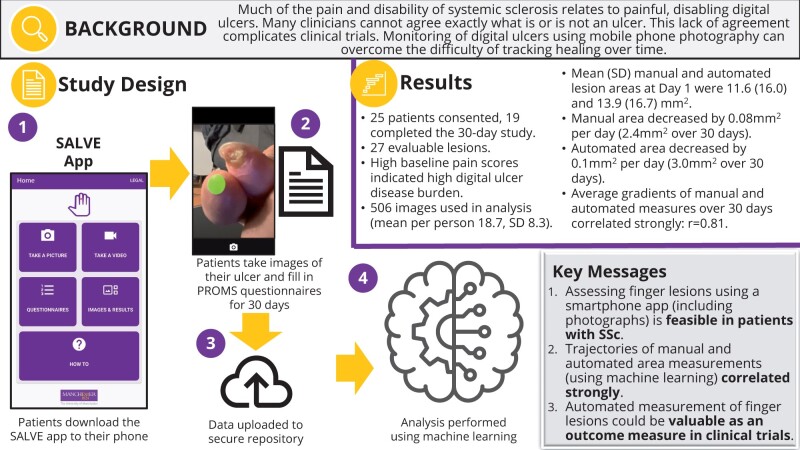

Rheumatology key messages

- Assessing finger lesions using a smartphone app (including photographs) is feasible in patients with SSc.
- Trajectories of manual and automated area measurements (using machine learning) correlated strongly.
- Automated measurement of finger lesions could be valuable as an outcome measure in clinical trials.

## Introduction

Much of the pain and disability of the CTD SSc (also termed ‘scleroderma’) relates to painful, disabling digital ulcers (ulcers of the fingers and sometimes toes) which develop in ∼50% of patients [1, 2]. These ulcers interfere with everyday activities and often make it impossible for those affected to continue working [3, 4]. Current treatments for digital ulcers are often only partly effective (if at all). Therefore, new treatments are required. Disappointingly, several recent multinational trials failed to meet their primary endpoint [5–7], despite many patients (and their clinicians) feeling that the drug being tested conferred benefit.

Many clinicians believe the reason that some of these recent studies were ‘negative’ was our inability to prove effectiveness, rather than the treatment itself being ineffective. A major barrier to developing and implementing effective treatments is that clinicians cannot agree on what is/is not an ulcer, and whether an ulcer has healed [8–12]. This inability to define and to measure ‘ulcers’ (which we shall refer to later as ‘lesions’ given the problems with terminology) is a very major problem. This is because in trials of treatment, the primary endpoint is usually the number of digital ulcers a patient develops during the trial, or the time it takes for the ulcers to heal. Reduction in ulcer size is rarely included. It is therefore hardly surprising that if proving effectiveness depends on something we struggle to measure, studies are bound to fail. Another problem is that even if healing could be accurately defined, study visits are typically 4 weeks apart [5], and so a lesion which healed at day 29 would be judged similarly to a lesion healing at day 55, although this is very different for the patient. As a result of these difficulties, pharmaceutical companies have been reluctant to fund further studies.

Mobile phone monitoring of digital lesions could overcome this difficulty [13]. Nowadays, most people carry a smartphone, providing the ideal platform to capture photographs/images of finger lesions repeatedly over time, with many patients bringing photographs of their lesions to the outpatient clinic [14], demonstrating how they recognize the value of photographic evidence.

Our overall aim was to test the hypothesis that data collected daily using a smartphone app designed specifically for digital lesions can provide an objective outcome measure, sensitive to change, for use in clinical trials. The first step, successfully completed [15], was to develop a robust, device-agnostic protocol for patients to capture images (i.e. photographs) of their own digital lesions using their own phones. The specific objectives of the current study were to:

- Develop a smartphone app that guides patients through the image capture process and that also captures non-imaging patient-reported outcome measures (PROMs), namely pain, hand function and ‘ulcer/lesion’ burden, and then test the feasibility of using this in patients with SSc and current digital lesions.
- Develop methods to extract and analyse all available data from the photographs (size and colour) in order to track healing status automatically, comparing manual measurement of lesion area (size) versus automated measurement, and examining associations between lesion area and pain.

## Methods

### Participants

Patients fulfilling the 2013 criteria for SSc [16] and with one or more current finger lesions were recruited. All were attending a single tertiary centre for SSc and all were over 18 years of age. Participants were recruited face-to-face or remotely, either because they attended hospital with a finger lesion, or because they responded to an invitation letter informing them to contact the hospital in the event of their developing a finger lesion. All participants signed written informed consent (South West—Cornwall and Plymouth Research Ethics Committee—REC reference 21/SW/0105). A patient could be re-recruited into the study if they developed a new lesion after completion of the initial 30-day study period. Each patient recruitment will be referred to as an ‘episode’.

#### Patient pathway through the study

Participants either used their own smartphone or (for those without a suitable handset) were lent one for the study. The app (described below) was loaded on to the phone using the app store relevant to the make of the smartphone (i.e. iPhone, Android). Participants collected data with the app daily over a period of ∼30 days and completed both a pre- and post-feedback study questionnaire. As described below, they were asked to take a photograph of their finger lesion(s) daily. They were alerted via a smartphone notification when and which PROMs questionnaires were to be completed. For example, a rating of pain (as described below) was required daily, so a notification appeared everyday around 18:00 h.

### App development

The app was co-designed with a patient user group during three meetings, attended by five patient advisers to the research team, all of whom had experienced SSc-related finger lesions. In addition to collecting images, the app was designed to capture non-imaging PROMs (see Supplementary Material), as decided upon during two of the user group meetings:

- Daily assessment of pain (Pain score), as assessed by a Likert scale 0–10 (10 = ‘worst possible pain’) using radio buttons.
- Weekly assessment of interference of daily activities by RP, by visual analogue scale (VAS) (Raynaud’s-VAS) scale 0–100 [100 = very severe limitation, similar for (iii) and (iv)].
- Weekly assessment of interference of daily activities by finger ulcers/lesions, by VAS (Finger lesion-VAS), scale 0–100.
- Weekly assessment of overall disease severity, by VAS (Disease severity-VAS), scale 0 to 100 [(ii), (iii) and (iv) were modified from the VAS of the Scleroderma Health Assessment Questionnaire (SHAQ)] [17].
- Assessment of hand disability [Hand Disability in Systemic Sclerosis-Digital Ulcers (HDISS-DU)] [18] at Day 1 (at entry into the study) and at day 30 (end of study). The HDISS-DU comprises 24 questions, each scored 0 to 5 (0 = yes, without difficulty; 5 = impossible) with additional boxes 6 and 7 (6 = did not do this activity in the past 7 days; 7 = used unaffected hand only).

**Figure 1.**
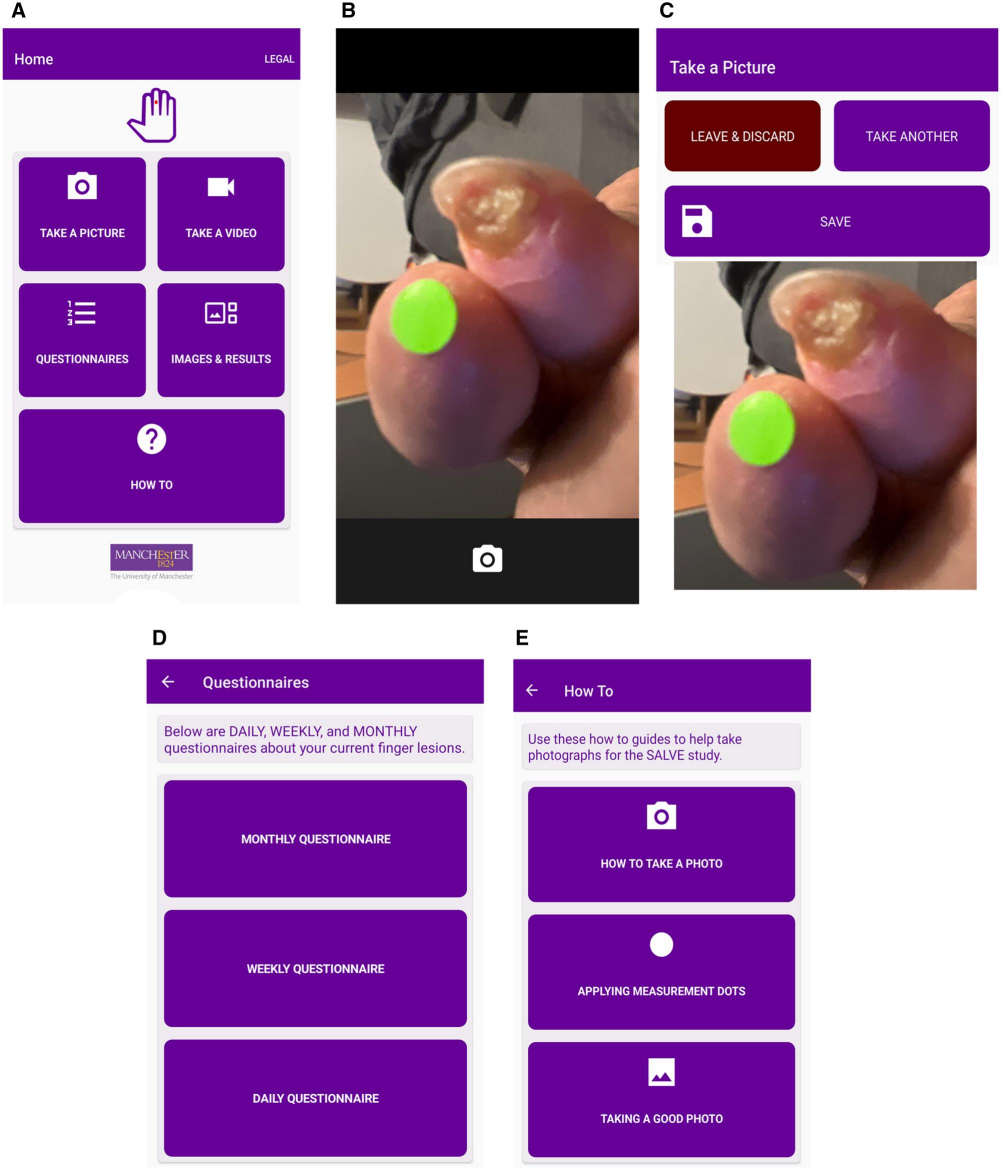
Screenshots from the SALVE (Scleroderma App for Lesion VErification) app. (**A**) The home page; (**B**) the live view while taking a photograph; (**C**) the preview of the taken photograph and the option to save, discard or take another; (**D**) the questionnaire selection page; and (**E**) the ‘how to’ page that informs users on how to use the app and its features.

VAS were completed by patients using a ‘slider’ that went from 0 to 100. The app was developed using Xamarin, an open-source app development platform that allowed creation of one code base but allowed deployment to both iOS and Android simultaneously. A primary feature of the app was a dedicated image (photo)-taking area for lesions, where a participant would take an image each day and submit to the research team (Fig. 1). After feedback from the patient user group, these images were not saved to the participant’s device to prevent filling up storage space. The second primary feature was digital questionnaires for the PROMs described above. Other general features included a how-to guide, interface testing and the legal documents to be accepted when a patient first used the app.

### Imaging protocol

The imaging protocol, and its development, are as previously described [15]: the protocol itself can be viewed in the Supplementary Material. In brief, participants were asked to photograph one or more lesions each day for 30 days, ideally at the same time each day and in the same location. Either the rear-facing (standard) or front-facing camera could be used: whichever the patient found easier. A 6 mm adhesive dot was placed adjacent to the lesion(s) to provide a reference scale. Participants uploaded images to a secure Dropbox folder.

### Assessing feasibility

Feasibility was assessed from a combination of patient feedback (post-study questionnaire), the number of images submitted (as a proportion of ‘ideal’ number expected within the timeframe of the study), and the number of pain scores submitted (also as a proportion of ‘ideal’).

### Extraction and analysis of imaging data from the app

Lesion area was analysed using ‘manual’ and ‘automated’ methods. For the manual method, borders between intact and lesional skin on all images were identified and annotated by a single observer (A.K.D.), giving a measurement of area.

To analyse the change in lesion colour with time, the area of the lesion selected was manually annotated in the first (i.e. earliest) image (including any noticeable boundaries where the skin had healed). This boundary was kept consistent across each timepoint in a patient’s image sequence, as we were interested in seeing the colour change back to healed skin (had the boundary been made smaller to follow the healing process, then no colour change would be measured). The colour of the area within this boundary was then calculated automatically across timepoints. We used L*a*b* colour space to analyse pixels within the annotated areas, a colour measurement system developed to replicate how the human eye perceives colour. L*a*b* space has three components: L* is the lightness, a* is the red/green values and b* is the blue/yellow values (each represented on one of three axes in 3D space). The colour spectrum in L*a*b* colour space is large, meaning that the very close shades of colour that can occur in lesions can be found as they would be distinguished by the human eye.

Automated measurements of lesion size were obtained using machine learning, namely by using segmentation. The model we used was trained and tested using images from our feasibility study of mobile phone photography [15], which included 332 images of 18 digital lesions. The training set of images were annotated by drawing a boundary around each lesion. These images were used to train a segmentation model using PointRend [19] as the segmentation approach, and this model was tested with a test set of images from the same image set. Once the model was trained, we could use all the new data from the current study to output new segmentation mask predictions, which allowed us to extract the lesion area. In a future study, these predicted masks could be used to analyse the colour using the same technique as the manual analysis used here, but without the need for manually drawing a boundary.

### Statistical analysis including sample size calculation

A sample size of 25–30 participants was chosen to capture diversity in participant behaviour and lesion appearance, and to ensure collection of a sufficiently large number of lesions to support a machine learning approach. Statistical power was not a consideration for this early-stage feasibility study.

Analysis was largely descriptive. A regression model was fitted to each participant’s individual area (manual and automated) data to calculate the average gradient over the 30-day study period. Missing images were categorized as ‘not submitted’ or ‘submitted but not usable’. Average size gradient was correlated (Pearson’s) with change in colour and change in pain score.

All statistical analysis was performed using Stata version 14.

## Results

**Figure 2.**
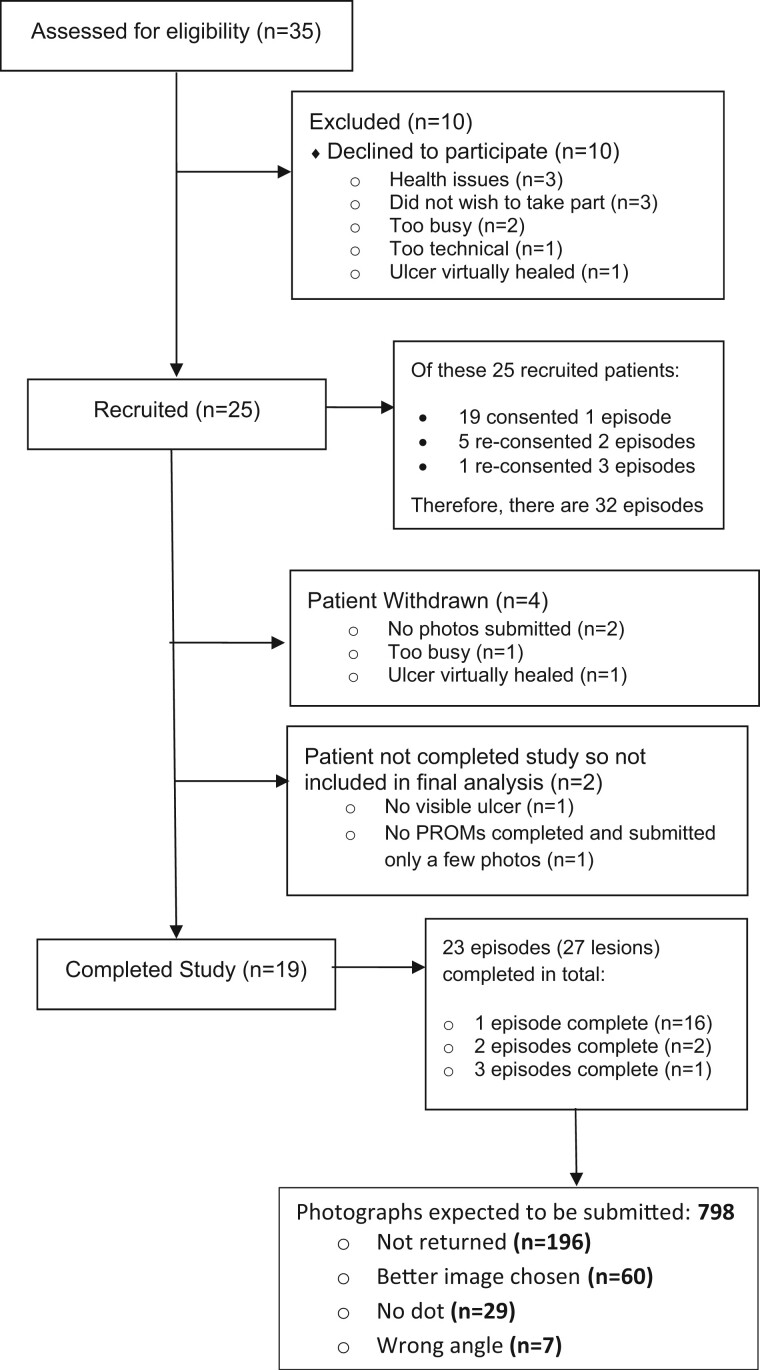
CONSORT (Consolidated Standards of Reporting Trials) flow diagram.

Patient flow through the study is summarized in Fig. 2. Of the 35 participants assessed for eligibility, 25 were recruited, of whom 19 consented for one episode, 5 for two episodes and 1 for three episodes (giving 32 episodes in total). Nineteen participants completed the study, with evaluable data from 23 episodes and 27 lesions (Fig. 2): four participants had two lesions within one episode. Of the six participants who did not complete the study, four withdrew, one did not submit any PROMs data (and only very few images) and in one participant the lesion had healed prior to images being submitted.

The clinical and demographic data of the 25 participants who submitted data are summarized in Supplementary Table S1 available at *Rheumatology* online. Their mean (s.d.) age was 57.6 (13.1) years. Twenty-two (88%) were female, 22 (88%) had the limited cutaneous subtype of SSc and 3 (12%) diffuse cutaneous disease. Mean (s.d.) duration of RP was 23.0 (14.1) years and of SSc (from date of onset of first non-Raynaud’s clinical manifestation) was 19.0 (11.0) years. Twenty-two (88%) were on vasodilator therapy and 9 (36%) were on bosentan. Seventeen (68%) had had previous i.v. prostanoid therapy for SSc-related digital vasculopathy and three (12%) received i.v. iloprost at the time of the study.

Results from the pre-study questionnaire, which asked participants about their mobile phone usage, indicated that 22 (88%) owned a mobile phone, 2 did not and one patient reported that his wife owned a phone. The two participants without access to a phone were lent one for the duration of the study. When asked if/how their finger lesion(s) led to impaired ability to use a mobile phone, 2 (8%) reported that they were unable to use a phone, 4 (16%) stated ‘a lot’ (of impairment), 16 (64%) ‘a little’ (impairment) and 3 (12%) no impairment.

### Feasibility of using the app

#### Completeness of submitted daily images and PROMs

The 19 participants were expected to return 798 images (30 images each for 26 lesions, and 18 images for 1 lesion as the patient withdrew). Further details on the breakdown of the number of photographs are given in the CONSORT (Consolidated Standards of Reporting Trials) (Fig. 2). In total, 506 images were used/usable out of a possible total of 542 (93%) from 27 lesions, and 474 pain scores out of a possible total of 678 (70%). Frequency of submission of images and pain scores for individual participants is summarized in Supplementary Fig. S1 available at *Rheumatology* online.

#### As assessed by post-study questionnaire

On a 1–10 scale (1 = very easy, 10 = very difficult), the median (range) responses to questions included: ‘Remembering to take photographs/videos of finger ulcers’ 1.5 (1–8); ‘Taking photographs/videos at the same time every day’ 2.5 (1–8); ‘Taking photographs/videos in the same place every day’ 1 (1–8); ‘Keeping the environment and lighting the same each time’ 1.5 (1–7); ‘Overall experience of using the mobile phone to photograph/video your finger ulcer’ 2.5 (1–10); ‘Holding the phone when imaging’ 5 (1–10); ‘Pressing the button or screen to take an image’ 3.5 (1–10); and ‘Getting a good clear image of your finger ulcer’ 5 (1–10). Six participants stated that they had help in acquiring the images. Only three patients took videos in addition to single frame photographs.

### Imaging data, and assessment of lesion area

Of the 27 lesions from which images were returned, 13 were fingertip lesions, 6 extensor lesions and 8 were at other locations on the finger.

**Figure 3.**
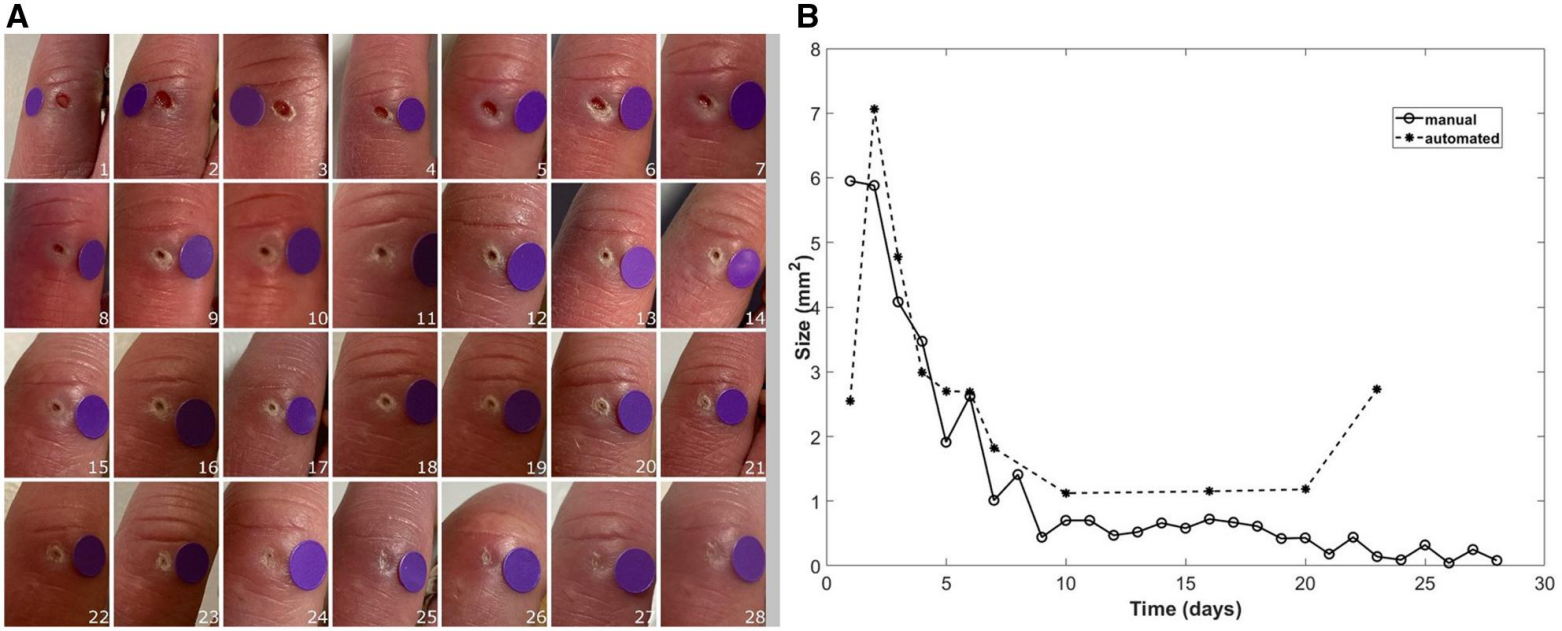
Exemplar series of images over 30 days. (**A**) Series of images from a lesion on the extensor aspect of a left little finger proximal interphalangeal joint (showing ulcer healing over the study period, numbers refer to study day). (**B**) Graph of change in manual and automated measurements over time. Some automated measurements were not captured due to the segmentation model ‘missing’ the ulcer (see text).

**Figure 4.**
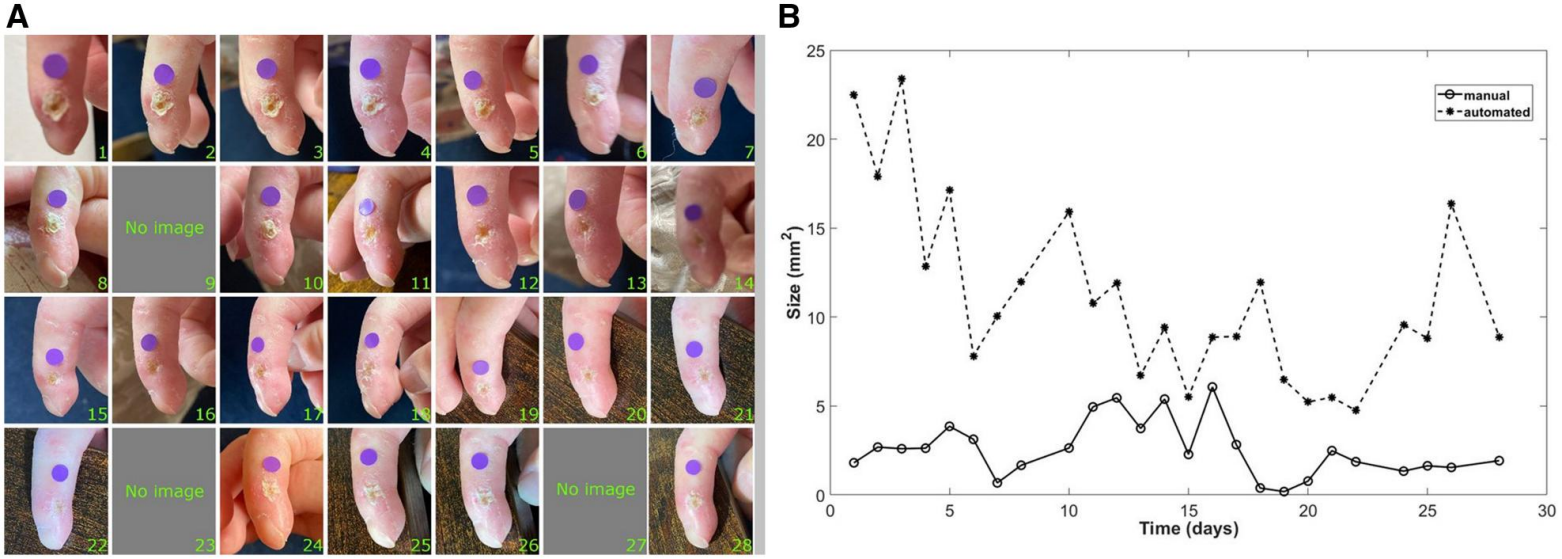
Exemplar series of images over 30 days. (**A**) Series of images from a lesion on the lateral aspect of a distal interphalangeal joint (showing ulcer healing over the study period, numbers refer to study day). (**B**) Graph of change in manual and automated measurements over time.

Of the 602 images returned, 506 (84%) were used in the analysis. Sixty (10%) were not used because a better image was chosen (some patients took more than one image from the same day), 29 (5%) because there was no dot included in the image and 7 (1%) because the photograph was taken at the wrong angle (Fig. 2). The mean number of used images per lesion was 18.7 (s.d. 8.3). Figs 3 and 4 (left-hand panels) show two examples of series of images from very different types of lesion, demonstrating change over time.

From the 506 images, 86 automated measurements were unavailable as the machine learning model could not detect a lesion: these 86 false-negative predictions tended to be where the lesion was very small or could not be easily distinguished from normal skin.

Baseline manual measurements were available from 26 lesions, with a mean lesion area of 11.6 mm^2^ (s.d. 16.0). Baseline automated measurements were available from 23 lesions with a mean of 13.9 mm^2^ (s.d. 16.7).

### Patient-reported outcome measures

Baseline PROM results (Pain score, Raynaud’s-VAS, Finger lesion-VAS, Disease severity-VAS and HDISS-DU) are summarized in Table 1, indicating that this was a group of participants with high lesion- and disease-related morbidity: mean Pain score at baseline was 5.7 (s.d. 2.4). PROM results recorded during the study are also summarized in Table 1. The mean daily Pain score throughout the study period was 5.2 (s.d. 2.7).

**Table 1.** PROMs data at baseline (day 1) and at day 8, day 15 and at end of study (day 29)

PROM	Day 1/baseline [mean (s.d.)]	Day 8/week 1 [mean (s.d.)]	Day 15 [mean (s.d.)]	Day 29/end of study [mean (s.d.)]
HDISS-DU	2.2 (0.9) (*n* = 14)			1.9 (0.9) (*n* = 14)
Pain score (scale 0–10)	5.7 (2.4) (*n* = 23)	6.0 (2.9) (*n* = 22)	5.0 (2.9) (*n* = 20)	5.0 (2.5) (*n* = 23)
VAS scores SHAQ (scale 0–100)				
Raynaud’s-VAS	70.7 (21.8) (*n* = 23)	62.6 (23.4) (*n* = 21)	61.7 (25.6) (*n* = 21)	57.0 (26.1) (*n* = 19)
Finger lesion-VAS	72.4 (22.1) (*n* = 23)	61.9 (27.0) (*n* = 21)	63.7 (27.5) (*n* = 21)	62.5 (26.8) (*n* = 19)
Disease severity-VAS	66.0 (21.0) (*n* = 23)	60.2 (22.9) (*n* = 21)	63.8 (26.0) (*n* = 21)	63.4 (26.0) (*n* = 19)

HDISS-DU: Hand Disability in Systemic Sclerosis-Digital Ulcers; PROM: patient-reported outcome measure; VAS: visual analogue scale; SHAQ: Scleroderma Health Assessment Questionnaire.

### Analysis of lesion area over 30-day study period

The results from the analysis of manual lesion measurements over the 30-day study period suggested that on average lesion area decreased by 0.08 mm^2^ per day for 30 days, i.e. decreasing by 2.4 mm^2^ over the 30-day study period.

The results from the analysis of automated lesion measurements over the 30-day study period, suggested that on average lesion area decreased by 0.1 mm^2^ per day for 30 days, i.e. decreasing by 3.0 mm^2^ over the 30-day study period. The right-hand panels of Figs 3 and 4 demonstrate examples of change over time in both manual and automated measurements in different types of lesion.

### Statistical comparison between manual and automated measurements

**Figure 5.**
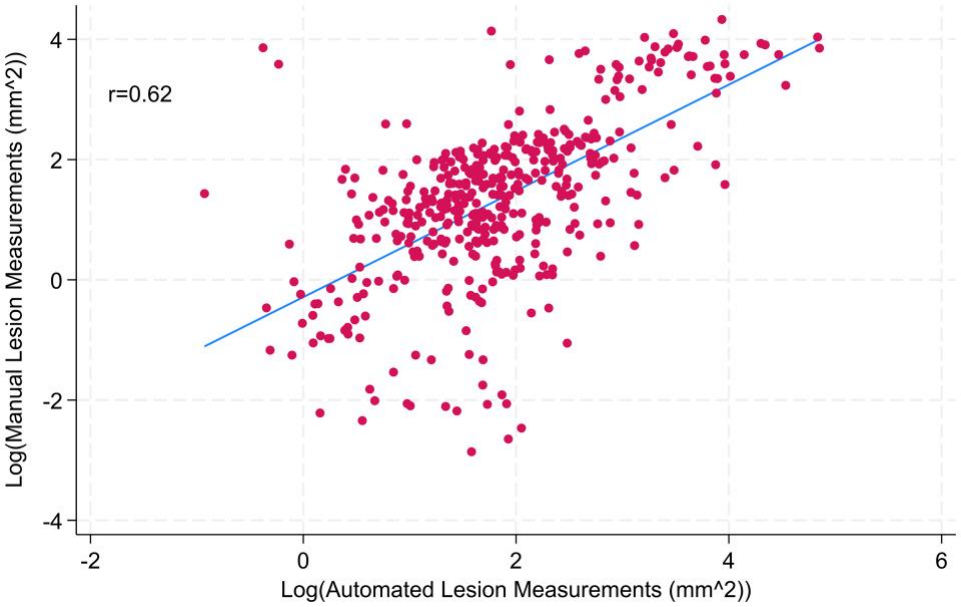
Scatterplot of manual lesion measurements against automated lesion measurements. There would have been perfect agreement between the two methods if all points in the scatterplot were on the line of equality. The line of equality is the straight line shown, with all points on the line having the same size of lesion for both methods. It is important to note that 86 measurements were missing for the automated measurements as the machine learning model was unable to evaluate them.

Fig. 5 shows the correlation of manual and automated measurements of the area of the lesion (r = 0.62) (*P* < 0.001).

The Bland–Altman plot (Supplementary Fig. S2 available at *Rheumatology* online) shows that the manual measurements were on average 40% lower than the automated measurements. The limits of agreement show that 95% of manual measurements were between 0.076 and 4.8 times those of automated measurements. This interval is wide, indicating that large differences in measurements were seen.

The correlation between the average gradients of manual and automated measurements over 30 days was 0.81 (Supplementary Fig. S3 available at *Rheumatology* online) (*P* < 0.001), suggesting a strong positive relationship between the gradients estimated from manual and automated measurements.

In the examples shown in Figs 3 and 4, manual and automated measurements of colour correlated reasonably well although with some discrepancies.

### Associations between change in lesion area, change in colour and change in pain

Supplementary Fig. S4 available at *Rheumatology* online shows the correlation between colour distribution gradient over time and area gradient (for both manual and automated measurements). The scatterplots suggest that there are no clear relationships between the manual or automated measurements of change in lesion area and change in lesion colour. Similarly, we assessed the associations between manual and automated size over time, and colour, with Pain score. Supplementary Fig. S5 available at *Rheumatology* online suggests that there is no clear response pattern between any of the three image-derived measures and change in Pain score.

## Discussion

The main findings of this study, which was informed throughout by patient opinion and experience, were that using the digital lesion app was feasible for patients with SSc, even in those with significant hand disability (as demonstrated by the number of images and PROMs returned, and favourable participant feedback) and that there was a strong positive relationship between average gradients of manual and automated measurements over the 30 day study period (examining change over time). This latter finding suggests internal consistency of the automated measurement that could make it valuable as an outcome measurement for most finger lesions. Automated measurements correlated with manual measurements, although limits of agreement were wide.

The app therefore has potential in monitoring outcome in clinical trials of SSc-related finger lesions, combining PROMs with objective ‘time-stamped’ visual assessment of lesion appearance, as exemplified in Figs 3 and 4. A major advantage is that app data can be collected remotely, without the need for hospital attendance. Some lesions were challenging to assess because of different lighting conditions and angles of view, and lesion position (e.g. at the nailbed).

We chose to assess both lesion area (size) and colour, believing that both of these could serve as indices of lesion healing. Colour proved especially challenging to measure. As exemplified in Figs 3 and 4, different skin characteristics contribute to colour and these may not always be related to lesion healing. Another challenge when assessing colour is that it is very dependent on lighting conditions. Although patients’ perceptions suggested that they felt they could successfully ensure similar lighting conditions each day, visual inspection of images suggested that lighting conditions could vary between consecutive images and could complicate interpretation of results.

Within the 30 day time frame of the study, lesions tended to improve in terms of both size and PROMs. We found no relationship between change in lesion area (or colour) and change in Pain score, although the relatively short study duration may have been a contributory factor. It is recognized that digital lesions often take several weeks (or even months) to heal [20].

Although our focus was development of an outcome measure, the COVID-19 pandemic provided additional impetus to examining mobile tools to monitor ulcer/wound healing in the clinical setting, in order to reduce the requirement for patients to attend hospital when many were reluctant to do so. Zhang *et al.* developed an mHealth tool which (similar to our app) allowed patients to image digital ulcers, and demonstrated feasibility in 15 patients with SSc [21]. Their study design included using a smartphone holder to facilitate image acquisition, and providing feedback on image quality. Smartphone-based applications have been used to assess and monitor wounds in other conditions including diabetic foot ulcers and post-surgical wounds [22, 23]. This is an area of active research.

Our study had limitations. First, the relatively small number of lesions studied, especially given the heterogeneity of digital lesions. Second, the assessment of manual size by one observer only. A next step will be to compare automated measurement with expert consensus opinion of manually measured area, including consideration of intra- and inter-observer variability. A third limitation related to our automated method: although the training images from our previous feasibility study [15] represented a large dataset of images for a rare disease, deep learning approaches, such as the one used in this study, perform best when more data are available to model the most variation. This concern most likely contributed to the large number of ‘false-negative’ automated measurements and to some of the discrepancies between manual and automated measures, for example at the beginning of the series shown in Fig. 3. However, in this study we have established proof-of-concept for automated measurement of patient-submitted images. Future studies will include a larger number of images of different sizes, allowing us to explore more fully the influence of lesion size on manual and automated assessment. If we can demonstrate that automated measurements correlate with expert consensus opinion (as opposed to a single observer, as this may reduce variability), possibly in combination with computer-assisted planimetry [24]; then this will be a major step forward in providing an objective measure with face validity which could then be used as an outcome measure in clinical trials.

Patients were advised not to attempt to photograph lesions if dressed, but this was a pragmatic approach rather than a study limitation.

In conclusion, we have developed an app for collection of finger lesion images and PROMs, and demonstrated feasibility of using the app amongst patients with SSc. The app could be applied in both clinical and research settings, including in the context of clinical trials. The next steps are to improve the automation, assess reliability and to apply the app in larger prospective studies in order to validate its use as an outcome measure.

## Supplementary Material

keae371_Supplementary_Data

## Data Availability

The Sponsor will share de-identified individual participant data collected during the trial with researchers who provide a methodologically sound proposal.
