# Supplementary material for: Development of a measuring app for systemic sclerosis-related digital ulceration (SALVE: Scleroderma App for Lesion VErification)
Source: Rheumatology (Oxford). 2024 Jul 19;63(12):3297–305. doi: 10.1093/rheumatology/keae371 (PMC11637552; doi:10.1093/rheumatology/keae371)
Supplement: keae371_Supplementary_Data [file keae371_supplementary_data.docx]

Supplementary material - Development of a measuring app for systemic sclerosis-related digital ulceration (SALVE: Scleroderma App for Lesion VErification)

Supplementary Table 1 - Demographics and clinical characteristics of the 25 participants with SSc recruited into the study

|  | **N** | Mean (Standard Deviation) or frequency (%) |
| --- | --- | --- |
| Age (Years) | 25 | 57.6 (13.1) |
| Gender |  |  |
| Male | 3 | 12% |
| Female | 22 | 88% |
| Disease sub-type |  |  |
| Limited | 22 | 88% |
| Diffuse | 3 | 12% |
| Disease duration (Years) from: |  |  |
| Date of onset of Raynaud’s | 25 | 23.0 (14.1) |
| Date of onset of first non- Raynaud’s manifestation | 25 | 19.0 (11.0) |
| Autoantibody status: |  |  |
| Anti-centromere |  |  |
| Positive | 15 | 60% |
| Anti RNA polymerase (n= 24) |  |  |
| Positive | 4 | 16% |
| Anti-Scl 70 |  |  |
| Positive | 5 | 20% |

Supplementary Figure 1 – Frequency distribution of Pain scores (submitted by 23 patients) and submitted images (images submitted by 23 patients from 27 lesions) over the 30 day study period.


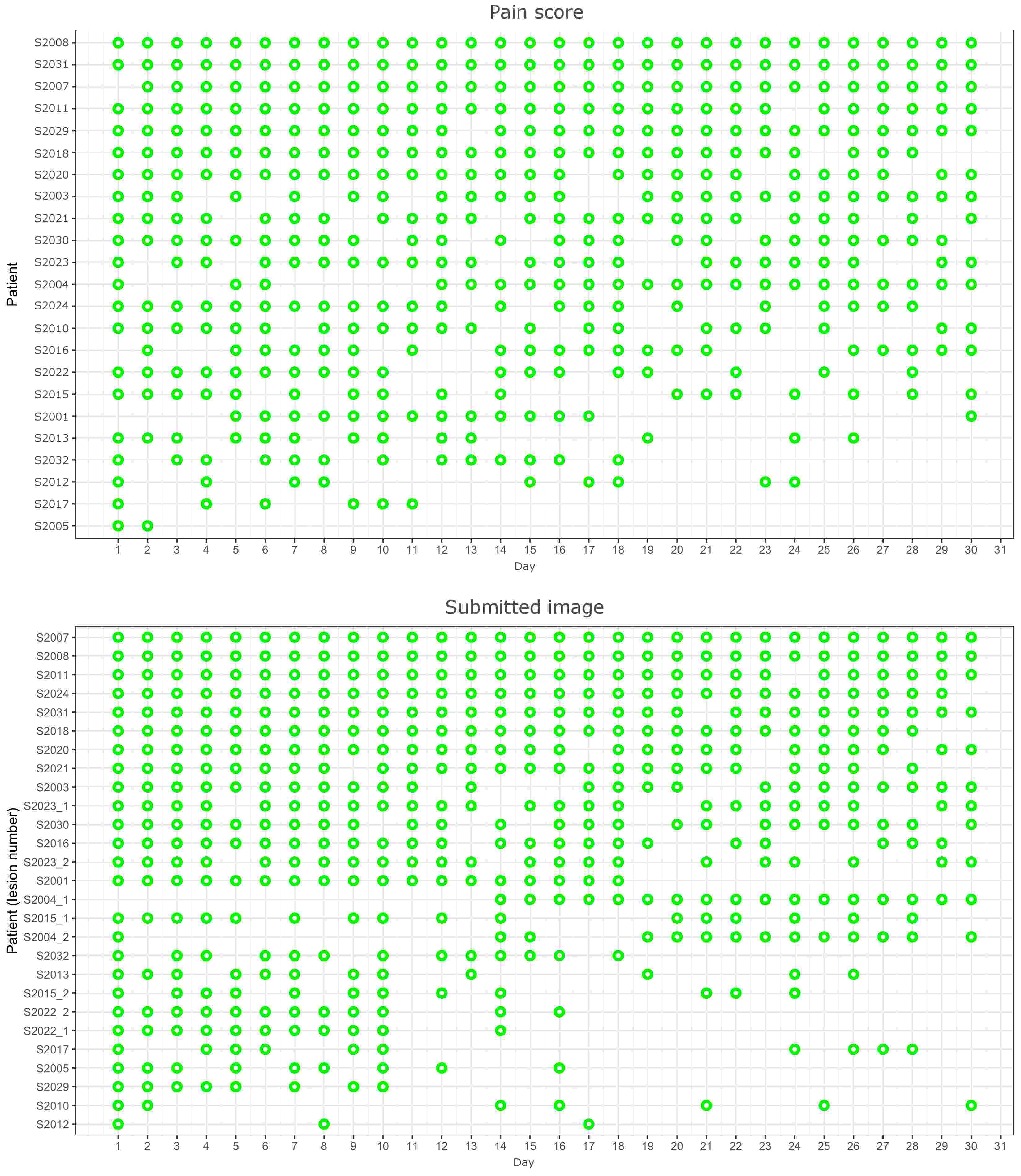


Supplementary Figure 2. Bland-Altman plot with 95% Limits of agreement using repeated measures. Each colour represents a different lesion.

Supplementary Figure 3. Scatterplot of average gradient of manual versus automated measurements.

Supplementary Figure 4: Scatterplots showing response patterns for (A) colour distribution gradient versus manual area gradient and (B) colour distribution gradient versus automated area gradient.

A.

B.

Supplementary Figure 5: Scatterplots of response patterns for image-derived measures versus Pain score. (A) Scatterplot showing response patterns for automated area gradient versus change in Pain score (B) Scatterplot showing response patterns for manual area gradient versus change in Pain score (C) Scatterplot showing response patterns for colour distribution gradient versus change in Pain score.

A.

B.

C.

Supplementary Figure 6: (A) Shows the outline from the manual annotation tool used to ‘draw’ the outline around a digital lesion (B) shows an example of a lesion (left) with the manual annotation overlayed (middle) and the corresponding automated annotation overlayed (right).

A.


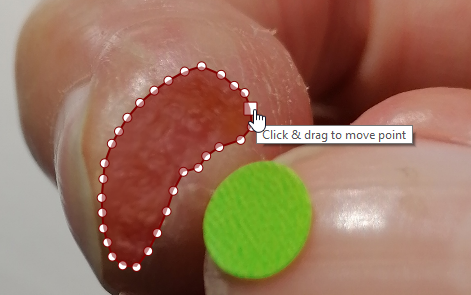


B.
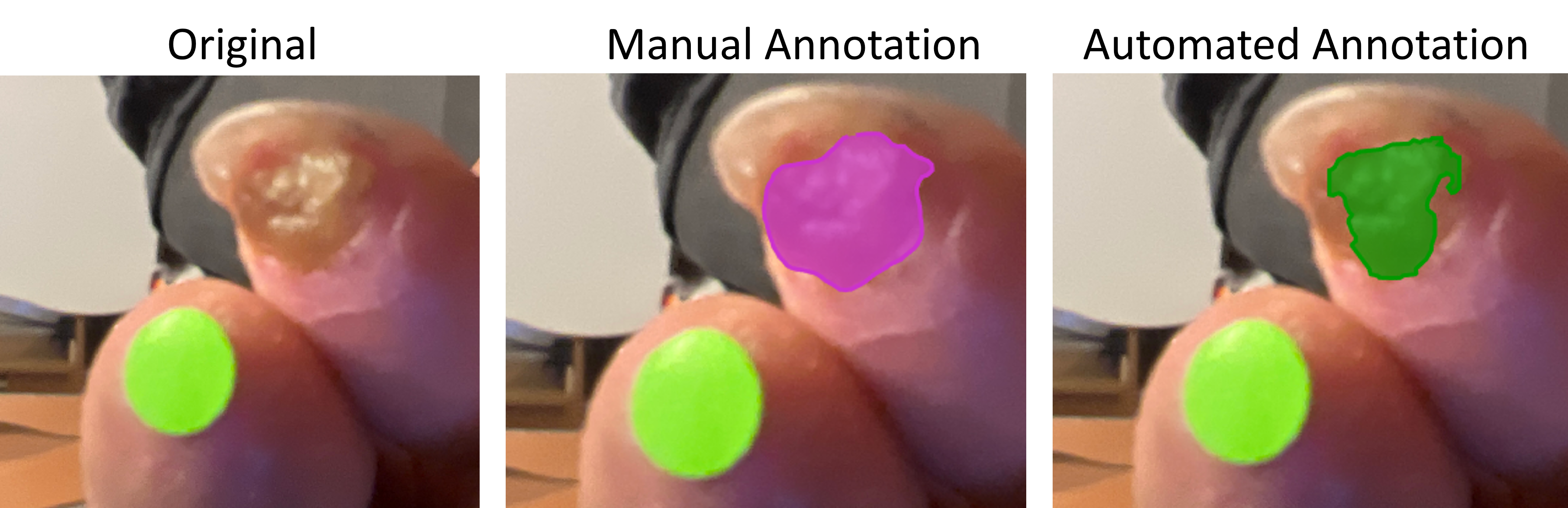


**Development of a measuring app for finger lesions as an outcome measure for systemic sclerosis-related digital ulceration – Study 2 (SALVE2: Scleroderma App for Lesion Verification 2)**

**Finger ulcer imaging protocol**

**Objective:** A simple set of instructions for patients to follow to allow them to capture high-quality photographs of their digital lesions (finger ulcers) using their smartphone camera and app.

**Scope:** For use by study participants testing a smartphone app as part of the SALVE study 2 (Scleroderma App for Lesion Verification 2) project.

**Assumptions:**

1. Participant has a smartphone with built-in camera and has had the SALVE study app installed onto the device.
2. Participant has one or more finger ulcers.

**Imaging timing and frequency:** participants will be asked to image each finger ulcer once per day for the duration of the study. It will be explained to them that this should ideally occur at the same time each day (e.g., just before bedtime). An app notification will alert the user when the next image is due.

**Environment:** the photographs/videos should, where possible, be taken in the same location every day. This is likely to be in the participants own home and should be consistently well lit and as free from visual clutter as is possible. It is recommended that there is a flat surface, such as a table or countertop on which the participant can rest their hands while imaging – this is to reduce the possibility of motion artefacts (blur). Keeping the background of the image in high contrast to the hand of interest is very important to ensure no hand features are lost or hard to identify. If a countertop, or similar is used, it should not have feature that could hinder analysis, such as household items or a woodgrain pattern.

**Considerations before imaging:** (to be discussed with the participant to take account of daily routines etc.) To help with consistency in imaging, it is best if the hands are in the same “state” at each imaging session. This should include time since last washed, application of hand creams or treatments, and removal or application of dressings on finger ulcers. If dressings are not to be removed on a particular day, then the participant can select “imaging not possible today” in the app, to skip the session.

**Taking photographs/videos:** Users have two options for photographing their finger ulcers: (1) is to use the rear (standard) camera on the phone, and photograph one hand while holding/operating the phone with the other; (2) is to use the front-facing camera (screen side/selfie camera) with the phone lying flat on a surface facing upwards. This second option is perhaps best for those participants with issues with hand function. Participants can use a ‘selfie stick’ or a tripod if this makes it easier for them to image their digital lesions, or if it helps them to steady the camera. Selfie sticks and tripods will be provided to participants by the research team upon request. Overall, the option will be up to the study participants.

Ideal lighting would be natural, with no bright glare and no shadows. However, this is hard to achieve without specific weather (such as overcast). It is recommended that participants should take two versions of a photograph: one with as best light as possible and the other using the phone’s flash. In addition, participants will be encouraged to record small videos (about 5 seconds) around the ulcer.

On opening the app, the user presses the “Take a Picture” button and is immediately taken to the live camera view. The user should position the camera and their finger ulcer in such a way that the ulcer is in clear focus, central to the frame, and can be held in a steady position. The user can then press the shutter button to take the image. Once the image is captured it can be accepted and sent to the appropriate approved storage or it can be retaken if the participant is unhappy with the picture preview shown.

For recording video, a similar process is taken. The user presses the “Take a Video” button and is taken to the video capture page, where videos can be recorded. The video should be captured from as many angles as possible, while keeping the finger ulcer central and in focus for the whole time. This section of the app will also allow users to upload videos they may have recorded outside of the app.

The participant repeats the process for each finger ulcer they wish to include in the study.

**Non-imaging Patient Reported Outcome Measures (PROMs)**

**MONTHLY questionnaire:**

**Hand Disability in Systemic Sclerosis – Digital Ulcers (HDISS-DU) Questionnaire**

**Thinking about using your hand(s) affected by ulcers on your fingers, please answer the questions below.**

**Please consider your ability to do these activities over the past 7 days.**

**If you did not complete the activity in the past 7 days, please check the box that says “Did not do this activity in the past 7 days.”**

**If you were only able to complete the activity by using an unaffected hand, please check the box that says “Used unaffected hand only.”**

1. Can you hold a plate full of food using your fingers?

| 🞎_0_ | 🞎_1_ | 🞎_2_ | 🞎_3_ | 🞎_4_ | 🞎_5_ | 🞎_6_ | 🞎_7_ |
| --- | --- | --- | --- | --- | --- | --- | --- |
| Yes, without difficulty | Yes, with a little difficulty | Yes, with some difficulty | Yes, with much difficulty | Nearly impossible to do | Impossible | Did not do this activity in the past 7 days | Used unaffected hand only |

1. Can you pour liquid from a large full bottle into a glass or cup using your fingers?

| 🞎_0_ | 🞎_1_ | 🞎_2_ | 🞎_3_ | 🞎_4_ | 🞎_5_ | 🞎_6_ | 🞎_7_ |
| --- | --- | --- | --- | --- | --- | --- | --- |
| Yes, without difficulty | Yes, with a little difficulty | Yes, with some difficulty | Yes, with much difficulty | Nearly impossible to do | Impossible | Did not do this activity in the past 7 days | Used unaffected hand only |

1. Can you use your bare hands to unscrew the lid from a jar that has previously been opened?

| 🞎_0_ | 🞎_1_ | 🞎_2_ | 🞎_3_ | 🞎_4_ | 🞎_5_ | 🞎_6_ | 🞎_7_ |
| --- | --- | --- | --- | --- | --- | --- | --- |
| Yes, without difficulty | Yes, with a little difficulty | Yes, with some difficulty | Yes, with much difficulty | Nearly impossible to do | Impossible | Did not do this activity in the past 7 days | Used unaffected hand only |

1. Can you cut food with a knife?

| 🞎_0_ | 🞎_1_ | 🞎_2_ | 🞎_3_ | 🞎_4_ | 🞎_5_ | 🞎_6_ | 🞎_7_ | |
| --- | --- | --- | --- | --- | --- | --- | --- | --- |
| Yes, without difficulty | Yes, with a little difficulty | Yes, with some difficulty | Yes, with much difficulty | Nearly impossible to do | Impossible | Did not do this activity in the past 7 days | | Used unaffected hand only |
|  |  |  |  |  |  |  | |  |

1. Can you use a fork to eat your food?

| 🞎_0_ | 🞎_1_ | 🞎_2_ | 🞎_3_ | 🞎_4_ | 🞎_5_ | 🞎_6_ | 🞎_7_ |
| --- | --- | --- | --- | --- | --- | --- | --- |
| Yes, without difficulty | Yes, with a little difficulty | Yes, with some difficulty | Yes, with much difficulty | Nearly impossible to do | Impossible | Did not do this activity in the past 7 days | Used unaffected hand only |

1. Can you prepare food with your bare hands?

| 🞎_0_ | 🞎_1_ | 🞎_2_ | 🞎_3_ | 🞎_4_ | 🞎_5_ | 🞎_6_ | 🞎_7_ | |
| --- | --- | --- | --- | --- | --- | --- | --- | --- |
| Yes, without difficulty | Yes, with a little difficulty | Yes, with some difficulty | Yes, with much difficulty | Nearly impossible to do | Impossible | Did not do this activity in the past 7 days | | Used unaffected hand only |

1. Can you wash dishes in the sink with your bare hands?

| 🞎_0_ | 🞎_1_ | 🞎_2_ | 🞎_3_ | 🞎_4_ | 🞎_5_ | 🞎_6_ | 🞎_7_ | |
| --- | --- | --- | --- | --- | --- | --- | --- | --- |
| Yes, without difficulty | Yes, with a little difficulty | Yes, with some difficulty | Yes, with much difficulty | Nearly impossible to do | Impossible | Did not do this activity in the past 7 days | | Used unaffected hand only |

1. Can you button your clothing?

| 🞎_0_ | 🞎_1_ | 🞎_2_ | 🞎_3_ | 🞎_4_ | 🞎_5_ | 🞎_6_ | 🞎_7_ | |
| --- | --- | --- | --- | --- | --- | --- | --- | --- |
| Yes, without difficulty | Yes, with a little difficulty | Yes, with some difficulty | Yes, with much difficulty | Nearly impossible to do | Impossible | Did not do this activity in the past 7 days | | Used unaffected hand only |

1. Can you open and close a zipper?

| 🞎_0_ | 🞎_1_ | 🞎_2_ | 🞎_3_ | 🞎_4_ | 🞎_5_ | 🞎_6_ | 🞎_7_ | |
| --- | --- | --- | --- | --- | --- | --- | --- | --- |
| Yes, without difficulty | Yes, with a little difficulty | Yes, with some difficulty | Yes, with much difficulty | Nearly impossible to do | Impossible | Did not do this activity in the past 7 days | | Used unaffected hand only |

1. Can you pull on your socks?

| 🞎_0_ | 🞎_1_ | 🞎_2_ | 🞎_3_ | 🞎_4_ | 🞎_5_ | 🞎_6_ | 🞎_7_ | |
| --- | --- | --- | --- | --- | --- | --- | --- | --- |
| Yes, without difficulty | Yes, with a little difficulty | Yes, with some difficulty | Yes, with much difficulty | Nearly impossible to do | Impossible | Did not do this activity in the past 7 days | | Used unaffected hand only |
|  |  |  |  |  |  |  | |  |

1. Can you tie your shoelaces?

| 🞎_0_ | 🞎_1_ | 🞎_2_ | 🞎_3_ | 🞎_4_ | 🞎_5_ | 🞎_6_ | 🞎_7_ | |
| --- | --- | --- | --- | --- | --- | --- | --- | --- |
| Yes, without difficulty | Yes, with a little difficulty | Yes, with some difficulty | Yes, with much difficulty | Nearly impossible to do | Impossible | Did not do this activity in the past 7 days | | Used unaffected hand only |
|  |  |  |  |  |  |  | |  |
|  |  |  |  |  |  |  | |  |

1. Can you hold a toothbrush to brush your teeth?

| 🞎_0_ | 🞎_1_ | 🞎_2_ | 🞎_3_ | 🞎_4_ | 🞎_5_ | 🞎_6_ | 🞎_7_ |
| --- | --- | --- | --- | --- | --- | --- | --- |
| Yes, without difficulty | Yes, with a little difficulty | Yes, with some difficulty | Yes, with much difficulty | Nearly impossible to do | Impossible | Did not do this activity in the past 7 days | Used unaffected hand only |

1. Can you wash your hands with soap and water?

| 🞎_0_ | 🞎_1_ | 🞎_2_ | 🞎_3_ | 🞎_4_ | 🞎_5_ | 🞎_6_ | 🞎_7_ |
| --- | --- | --- | --- | --- | --- | --- | --- |
| Yes, without difficulty | Yes, with a little difficulty | Yes, with some difficulty | Yes, with much difficulty | Nearly impossible to do | Impossible | Did not do this activity in the past 7 days | Used unaffected hand only |

1. Can you wash yourself in the shower or bath with your bare hands?

| 🞎_0_ | 🞎_1_ | 🞎_2_ | 🞎_3_ | 🞎_4_ | 🞎_5_ | 🞎_6_ | 🞎_7_ |
| --- | --- | --- | --- | --- | --- | --- | --- |
| Yes, without difficulty | Yes, with a little difficulty | Yes, with some difficulty | Yes, with much difficulty | Nearly impossible to do | Impossible | Did not do this activity in the past 7 days | Used unaffected hand only |

1. Can you brush your hair?

| 🞎_0_ | 🞎_1_ | 🞎_2_ | 🞎_3_ | 🞎_4_ | 🞎_5_ | 🞎_6_ | 🞎_7_ |
| --- | --- | --- | --- | --- | --- | --- | --- |
| Yes, without difficulty | Yes, with a little difficulty | Yes, with some difficulty | Yes, with much difficulty | Nearly impossible to do | Impossible | Did not do this activity in the past 7 days | Used unaffected hand only |

1. Can you put cream or lotion on your face with your bare hands?

| 🞎_0_ | 🞎_1_ | 🞎_2_ | 🞎_3_ | 🞎_4_ | 🞎_5_ | 🞎_6_ | 🞎_7_ |
| --- | --- | --- | --- | --- | --- | --- | --- |
| Yes, without difficulty | Yes, with a little difficulty | Yes, with some difficulty | Yes, with much difficulty | Nearly impossible to do | Impossible | Did not do this activity in the past 7 days | Used unaffected hand only |
|  |  |  |  |  |  |  |  |

1. Can you write a short sentence with a pencil or pen?

| 🞎_0_ | 🞎_1_ | 🞎_2_ | 🞎_3_ | 🞎_4_ | 🞎_5_ | 🞎_6_ | 🞎_7_ |
| --- | --- | --- | --- | --- | --- | --- | --- |
| Yes, without difficulty | Yes, with a little difficulty | Yes, with some difficulty | Yes, with much difficulty | Nearly impossible to do | Impossible | Did not do this activity in the past 7 days | Used unaffected hand only |

1. Can you use a keyboard to type?

| 🞎_0_ | 🞎_1_ | 🞎_2_ | 🞎_3_ | 🞎_4_ | 🞎_5_ | 🞎_6_ | 🞎_7_ |
| --- | --- | --- | --- | --- | --- | --- | --- |
| Yes, without difficulty | Yes, with a little difficulty | Yes, with some difficulty | Yes, with much difficulty | Nearly impossible to do | Impossible | Did not do this activity in the past 7 days | Used unaffected hand only |

1. Can you use your finger tips to press buttons on household appliances?

| 🞎_0_ | 🞎_1_ | 🞎_2_ | 🞎_3_ | 🞎_4_ | 🞎_5_ | 🞎_6_ | 🞎_7_ |
| --- | --- | --- | --- | --- | --- | --- | --- |
| Yes, without difficulty | Yes, with a little difficulty | Yes, with some difficulty | Yes, with much difficulty | Nearly impossible to do | Impossible | Did not do this activity in the past 7 days | Used unaffected hand only |

1. Can you use your finger tips to press small buttons, such as those on a cell phone?

| 🞎_0_ | 🞎_1_ | 🞎_2_ | 🞎_3_ | 🞎_4_ | 🞎_5_ | 🞎_6_ | 🞎_7_ |
| --- | --- | --- | --- | --- | --- | --- | --- |
| Yes, without difficulty | Yes, with a little difficulty | Yes, with some difficulty | Yes, with much difficulty | Nearly impossible to do | Impossible | Did not do this activity in the past 7 days | Used unaffected hand only |

1. Can you turn a key in a lock?

| 🞎_0_ | 🞎_1_ | 🞎_2_ | 🞎_3_ | 🞎_4_ | 🞎_5_ | 🞎_6_ | 🞎_7_ |
| --- | --- | --- | --- | --- | --- | --- | --- |
| Yes, without difficulty | Yes, with a little difficulty | Yes, with some difficulty | Yes, with much difficulty | Nearly impossible to do | Impossible | Did not do this activity in the past 7 days | Used unaffected hand only |

1. Can you use scissors to cut something?

| 🞎_0_ | 🞎_1_ | 🞎_2_ | 🞎_3_ | 🞎_4_ | 🞎_5_ | 🞎_6_ | 🞎_7_ |
| --- | --- | --- | --- | --- | --- | --- | --- |
| Yes, without difficulty | Yes, with a little difficulty | Yes, with some difficulty | Yes, with much difficulty | Nearly impossible to do | Impossible | Did not do this activity in the past 7 days | Used unaffected hand only |
|  |  |  |  |  |  |  |  |

1. Can you pick up coins using your finger tips?

| 🞎_0_ | 🞎_1_ | 🞎_2_ | 🞎_3_ | 🞎_4_ | 🞎_5_ | 🞎_6_ | 🞎_7_ |
| --- | --- | --- | --- | --- | --- | --- | --- |
| Yes, without difficulty | Yes, with a little difficulty | Yes, with some difficulty | Yes, with much difficulty | Nearly impossible to do | Impossible | Did not do this activity in the past 7 days | Used unaffected hand only |

1. Can you sweep the floor with a broom?

| 🞎_0_ | 🞎_1_ | 🞎_2_ | 🞎_3_ | 🞎_4_ | 🞎_5_ | 🞎_6_ | 🞎_7_ |
| --- | --- | --- | --- | --- | --- | --- | --- |
| Yes, without difficulty | Yes, with a little difficulty | Yes, with some difficulty | Yes, with much difficulty | Nearly impossible to do | Impossible | Did not do this activity in the past 7 days | Used unaffected hand only |

**Weekly questionnaire:**

**SHAQ Questionnaire**

IN THE PAST WEEK, how much have your finger ulcers interfered with your daily activities?

0 - 100, where 0 means the finger ulcers do not limit activities, and 100 is very severe limitations.

IN THE PAST WEEK, how much has Raynaud's interfered with your daily activities?

0 - 100, where 0 means the Raynaud's do not limit activities, and 100 is very severe limitations.

Overall, considering how much pain, discomfort, limitations in your daily life and other changes in your body and life, how severe would you rate your disease today?

0 - 100, where 0 means the finger ulcers do not limit activities, and 100 is very severe limitations.

**Daily questionnaire:**

**Pain Questionnaire**

Please rate your pain severity in the past 24 hours from 0 – 10, where 0 is no pain and 10 is the worst pain possible
